# Analysis of Seroma Formation, Flap Necrosis, and Postoperative Pain Following Modified Radical Mastectomy in Patients With Breast Cancer: A Prospective Study

**DOI:** 10.7759/cureus.94883

**Published:** 2025-10-18

**Authors:** Abhishek Ashok, Ashish K Chaudhary, Prem Shanker, Chandra S Singh, Arti, Yamini Rana

**Affiliations:** 1 Department of General Surgery, Maulana Azad Medical College, New Delhi, IND; 2 Department of Surgery, Ganesh Shankar Vidyarthi Memorial (GSVM) Medical College, Kanpur, IND; 3 Department of General Surgery, Ganesh Shankar Vidyarthi Memorial (GSVM) Medical College, Kanpur, IND; 4 Department of Anaesthesiology, Ganesh Shankar Vidyarthi Memorial (GSVM) Medical College, Kanpur, IND; 5 Department of General Anatomy, Ganesh Shankar Vidyarthi Memorial (GSVM) Medical College, Kanpur, IND

**Keywords:** breast cancer, flap necrosis, modified radical mastectomy (mrm), postoperative pain, seroma formation

## Abstract

Background

Breast cancer is one of the leading malignancies among Indian women, and it remains a significant contributor to the global cancer burden. Modified radical mastectomy (MRM), which involves removing the entire breast, nipple-areola complex, and axillary lymph nodes, is commonly performed. However, postoperative complications such as seroma formation, pain, and flap necrosis significantly impact patient recovery and quality of life.

Objective

This study aimed to evaluate the prevalence and presentation of seroma and flap necrosis, analyze the severity and distribution patterns of post-MRM pain, and assess associations between these complications and clinical parameters.

Methods

This prospective cohort study was conducted at a single tertiary-care center, Department of General Surgery, Ganesh Shankar Vidyarthi Memorial (GSVM) Medical College, Kanpur, India, from December 2022 to November 2024. One hundred consecutive patients with histologically confirmed breast carcinoma who were planned for MRM were included; patients aged <18 years, with bilateral disease, prior axillary surgery, prior chest wall radiation, local recurrence, metastatic disease, or undergoing reconstruction or breast-conserving surgery were excluded. Postoperative pain was measured using the validated Visual Analog Scale (VAS) on postoperative days (PODs) 7, 15, 30, 60, and 90. Seroma (assessed on POD 14) and flap necrosis were recorded by clinical examination using predefined surgical criteria. Data were collected using a standardized questionnaire and analyzed with descriptive statistics; Chi-square tests were used for categorical associations, and p < 0.05 was considered significant.

Results

Of 100 patients, 20 (20%) developed flap necrosis, and 26 (26%) experienced seroma. Age showed a significant association with pain severity on POD 7 (χ² = 18.487, p < 0.05) and seroma occurrence (χ² = 7.894, p < 0.05). BMI was strongly associated with seroma formation (χ² = 20.252, p < 0.05). Cancer stage correlated significantly with flap necrosis (χ² = 16.715, p < 0.05). Patients with seroma reported higher pain levels on POD 15 (p < 0.05), POD 30 (p < 0.05), and POD 90 (p < 0.05).

Conclusion

The findings emphasize the importance of patient-specific risk factor evaluation, including age, BMI, and cancer stage, to prevent complications and optimize postoperative care strategies. Early intervention and improved surgical techniques are essential to minimize seroma, flap necrosis, and pain severity after MRM.

## Introduction

Breast cancer continues to pose a major global health challenge. With an anticipated 665,255 female deaths from breast carcinoma in 2022, breast cancer was the most common cancer globally, according to data from the Global Cancer Observatory, International Agency for Research on Cancer (IARC)-WHO (2022). With an estimated 98,337 female breast cancer deaths in the same year, India had one of the highest national burdens [1,2]. In 2022, 670,000 fatalities worldwide were linked to breast cancer, which affected 2.3 million women. The mortality rate for women with breast cancer is 1 in 71, and the lifetime probability of receiving a diagnosis is 1 in 12 in countries with a relatively high Human Development Index (HDI) [1,2]. Breast cancer is the second most common malignancy among Indian women, accounting for 7% of the global burden [1,2]. The incidence of breast carcinoma varies by region, with the lowest rates in Africa and Asia, and the highest in North America and Europe. Only 0.5%-1% of cases occur in men, with over 99% affecting women, who are at a higher risk [1,2].

The WHO Global Breast Cancer Initiative (GBCI) aims to prevent 2.5 million breast cancer deaths globally between 2020 and 2040 by reducing the global breast cancer mortality rate by 2.5% annually [3]. Achieving this target would result in a 25% reduction in deaths by 2030, and, for women under 70, a 40% reduction by 2040 [3]. The three primary strategies of the initiative are to ensure comprehensive breast cancer management, enable prompt diagnosis, and promote early detection through health education [3].

Symptoms of breast cancer include a thickening or lump in the breast, which is frequently painless; changes in the size, shape, or appearance of the breast; and abnormalities in the overlying skin, such as dimpling, redness, or pitting. These may be accompanied by alterations in the appearance of the areola around the nipple, as well as unusual or bloody discharge from the nipple [4]. A substantial increase in the risk of breast cancer is caused by certain hereditary gene mutations, including BRCA1, BRCA2, and PALB2. Women who carry these genes might consider prophylactic mastectomy or chemotherapy as preventative interventions [4]. A commonly performed surgical technique for treating breast carcinoma is modified radical mastectomy (MRM), which involves removing the entire breast, including the nipple-areola complex and axillary lymph nodes [5]. Postoperative complications such as seroma formation, pain, and flap necrosis continue to pose serious threats to patient recovery and quality of life [5].

All these complications contribute to increased morbidity by prolonging hospital stay, delaying the initiation of adjuvant therapy, and adding to patients’ financial and psychological burden. This study was designed to provide a comprehensive analysis of postoperative complications following MRM, specifically focusing on pain, seroma formation, and flap necrosis. It evaluates the prevalence, presentation, and severity of these complications; examines patterns and distribution of post-MRM pain; and explores associations between seroma formation, flap necrosis, and various clinical parameters such as age, BMI, lymph node status, and disease stage. By addressing these relationships, the study seeks to identify risk factors that predispose patients to adverse postoperative outcomes. This work aims to fill a critical gap in the existing literature by providing detailed insight into these common, yet under-characterized, complications of MRM. The findings are intended to guide surgeons and healthcare providers in adopting evidence-based preventive strategies, refining surgical and perioperative practices, and ultimately contributing to the development of standardized protocols that enhance the safety, efficacy, and overall outcomes of MRM.

A pivotal shift toward breast conservation surgery occurred with a National Cancer Institute study that found no difference in survival rates between lumpectomy with radiation and mastectomy for Stage I/II breast cancer. As a result, the NIH 10 Consensus Conference in 1990 recommended breast conservation as the preferred treatment for early-stage breast cancer. Sentinel node biopsy has become a crucial focus in breast cancer staging. Recent studies, including the ACOSOG and NSABP B-32 trials, state that if sentinel lymph node biopsy (SLNB) is negative, SLNB surgery alone - with no further axillary lymph node dissection (ALND) - is appropriate, safe, and effective for breast cancer patients with clinically negative lymph nodes [6].

The most frequent early consequence of an MRM is a seroma, which is an accumulation of serous fluid in the dead space of the breast, axilla, or post-mastectomy skin flap. This can lengthen hospital stays, delay healing, and raise medical costs [7,8]. Although the exact cause of seroma production is unknown, it is thought to result from extensive soft tissue dissection that disrupts lymphatic and vascular drainage, causing an accumulation of serum and inflammatory exudate in the dead space. This can result in discomfort and other local wound problems, such as wound dehiscence. Incidence rates range from 10% to 85%, and risk factors include the extent of surgery, use of electrocautery, patient age, BMI, and comorbidities like diabetes and hypertension [7,8]. The degree of lymph node clearing, the quantity of positive nodes, the use of postoperative radiation, and whether intraoperative lymphatic channel ligation was carried out are some of the variables that influence seroma formation.

To prevent seroma formation, strategies such as utilizing suction drains, quilting sutures, fibrin sealants, and modifications in surgical technique can be employed. Research indicates that quilting sutures and drains significantly reduce both the incidence and volume of seromas. If seromas do develop, management typically involves aspiration and compression dressings. Minimal seromas do not cause any pain, deficit in function, or strain on the closure of the wound, and can be conservatively managed with observation. However, large-volume seromas - more than 75 to 100 mL - are associated with pain, infection, and reduced function of the shoulder joint movement in seromas of the axilla. Aspiration may be carried out in such patients. Debridement and open surgical drainage are sometimes necessary and are typically performed in chronic instances with encapsulated pseudocysts or infections [7,8].

Up to 60% of MRM patients report experiencing discomfort in the breast, chest wall, or arm after breast and axillary surgery, which is referred to as post-mastectomy pain. This type of pain is frequently described as hot, burning, sharp, or stabbing, and may occasionally be caused by nerve damage (neuropathic pain) [9]. Patients may also experience numbness, intermittent stabbing, dull discomfort, or sensitivity. It is impacted by the degree of tissue dissection, nerve damage, and pain threshold [9]. After MRM, pain is a complicated matter that depends on a number of parameters, including the degree of surgery, nerve injury, and the unique characteristics of each patient. Sufficient control over pain is essential, since insufficient pain management can result in persistent pain, decreased mobility, and a longer recovery time. Axillary web syndrome, commonly known as cording, affects around 10% of individuals following axillary dissection. There is a firm, painful structure that feels - and occasionally appears - as a cable in the armpit, frequently running down the arm, which limits mobility and necessitates management by a physiotherapist or lymphedema therapist [9]. Scar tissue formation and nerve injury from bruising, straining, or direct damage to the nerves during surgery are frequently blamed for persistent pain or discomfort. Pain sensations originating from the excised breast and nipple characterize phantom breast and nipple pain, a kind of nerve injury. These sensations can include pressure, itching, throbbing, or pins and needles. This imaginary pain may appear soon after surgery, or it may take a year or longer [9,10].

Multimodal analgesia, which includes opioids, non-steroidal anti-inflammatory drugs (NSAIDs), and local anesthetics, is frequently used in effective pain management, which is crucial for healing and quality of life. Techniques for regional anesthesia, such as thoracic epidurals and paravertebral blocks, have been shown to be successful in lowering pain and opioid consumption. A small percentage of patients experience chronic pain, also known as post-mastectomy pain syndrome (PMPS), which necessitates early intervention with physical therapy, counseling, and medication [10]. Studies show that radiation therapy and surgery for breast cancer can cause musculoskeletal pain, which typically manifests as shoulder joint restriction or as widespread pain and inflammation in particular muscle groups after treatment. After an MRM, specific exercises can help with the rehabilitation of shoulder and arm mobility. Numerous strategies have been demonstrated to help reduce this pain, including medication for pain management, focused physical therapy, and exercise [11].

Of MRM patients, 5% to 15% develop flap necrosis, or the loss of skin flaps as a result of inadequate blood flow. Comorbidities of the patient, smoking, surgical technique, and blood supply to the flap are among the contributing factors. This condition can cause delays in adjuvant therapy, along with poor aesthetics due to distortion, scarring, and increased risk of infection and implant extrusion, leading to psychological morbidity in the patient through anxiety and distress, and ultimately resulting in increased financial expenditure [12]. Patient risk factors for skin flap necrosis include advanced age, hypertension, diabetes, obesity, scarring from prior radiation treatment, smoking, and enlarged breast volume. Evaluation of skin color, dermal bleeding, skin temperature, and capillary refill time are all factors in determining the viability of a skin flap after MRM. Perfusion is measured using instruments such as indocyanine green angiography and intraoperative laser Doppler flowmetry [13].

Recommendations from the European Society of Medical Oncology and the National Institute for Health and Care Excellence (NICE) state that adjuvant therapy should begin within 31 days of the conclusion of surgery, and that treatment should ideally begin within two to four weeks [14]. The treating surgeon may, however, be reluctant to use radiation or chemotherapy when flap necrosis has affected or delayed wound healing [14]. Care should be taken not to overthin the flaps and risk flap necrosis. The oncoplastic plane that separates the subcutaneous fat tissue from the breast parenchyma can be followed to achieve this. The thickness of subcutaneous tissue varies greatly and is not correlated with a patient’s age, BMI, or the thickness in the contralateral breast. Surgical care involves excising the necrotic tissue and replacing lost skin in several ways: re-suturing, replacing lost skin with flaps or grafts, converting to an alternative breast reconstruction when needed, or allowing healing by secondary intention [13]. In cases of more widespread necrosis, vacuum dressings and other wound therapy instruments may help accelerate the healing process. Nonoperative management involves removing small pieces of eschar and applying dressings dynamically based on the appearance of the wound, such as alginates and silver preparations, to reduce the bacterial burden [15].

## Materials and methods

Study design and ethical approval

This prospective cohort study was conducted in the Department of General Surgery, Associate LLR Hospitals, General Surgery, Ganesh Shankar Vidyarthi Memorial (GSVM) Medical College, Kanpur, India (a single tertiary care center), over 1 year and 11 months (December 2022 to November 2024). Ethical approval was obtained from the Institutional Ethics Committee (Ref: EC/BMHR/2022/166; December 30, 2022), and written informed consent was obtained from all participants prior to enrolment. Consecutive patients aged ≥18 years, with histologically confirmed carcinoma of the breast who were planned for MRM, were screened and recruited. Patients were followed during their hospital stay until discharge, and subsequently in outpatient clinics for up to three months.

Operator experience and standardization

All MRMs were performed by a single consultant-led breast surgery team to minimize operator-related variability. Consultant surgeons with experience in breast oncology led every procedure, while trainees assisted under direct supervision when required. Operative technique, perioperative care, and documentation practices were standardized across the team.

Data collection, variables, and outcome definitions

Demographic and clinical variables recorded included age (years - later grouped as <30, 30-50, 51-70, >70); BMI (calculated from measured weight and height, and categorized as <25 vs ≥25); sex; side of disease (left/right); lymph node status (histopathology: positive/negative); TNM stage (I-IV); and neoadjuvant chemotherapy (NACT: yes/no). NACT was recorded as an exposure variable for exploratory and descriptive subgroup analyses only. Because this is an observational study, comparisons between patients who received NACT and those who underwent upfront surgery are reported as associations and should not be interpreted as causal comparisons.

Primary outcomes were postoperative pain, seroma formation, and flap necrosis. Pain severity was measured using the Visual Analog Scale (VAS; 0-10) on postoperative days (PODs) 7, 15, 30, 60, and 90, and categorized as mild (1-3), moderate (4-6), and severe (7-10). Pain character (localized vs. radiating) and nature (dull vs. sharp) were recorded from patient report and clinical examination. Postoperative analgesia was provided according to the institutional multimodal analgesia protocol and recorded in the case proforma. In brief, the standard regimen included scheduled paracetamol and an NSAID (unless contraindicated), with short-term opioid rescue (oral/IV) for breakthrough severe pain; regional blocks were not part of routine practice. Analgesic agents, dosages, and any modifications were documented at each follow-up visit and considered when interpreting VAS scores.

**Figure 1:**
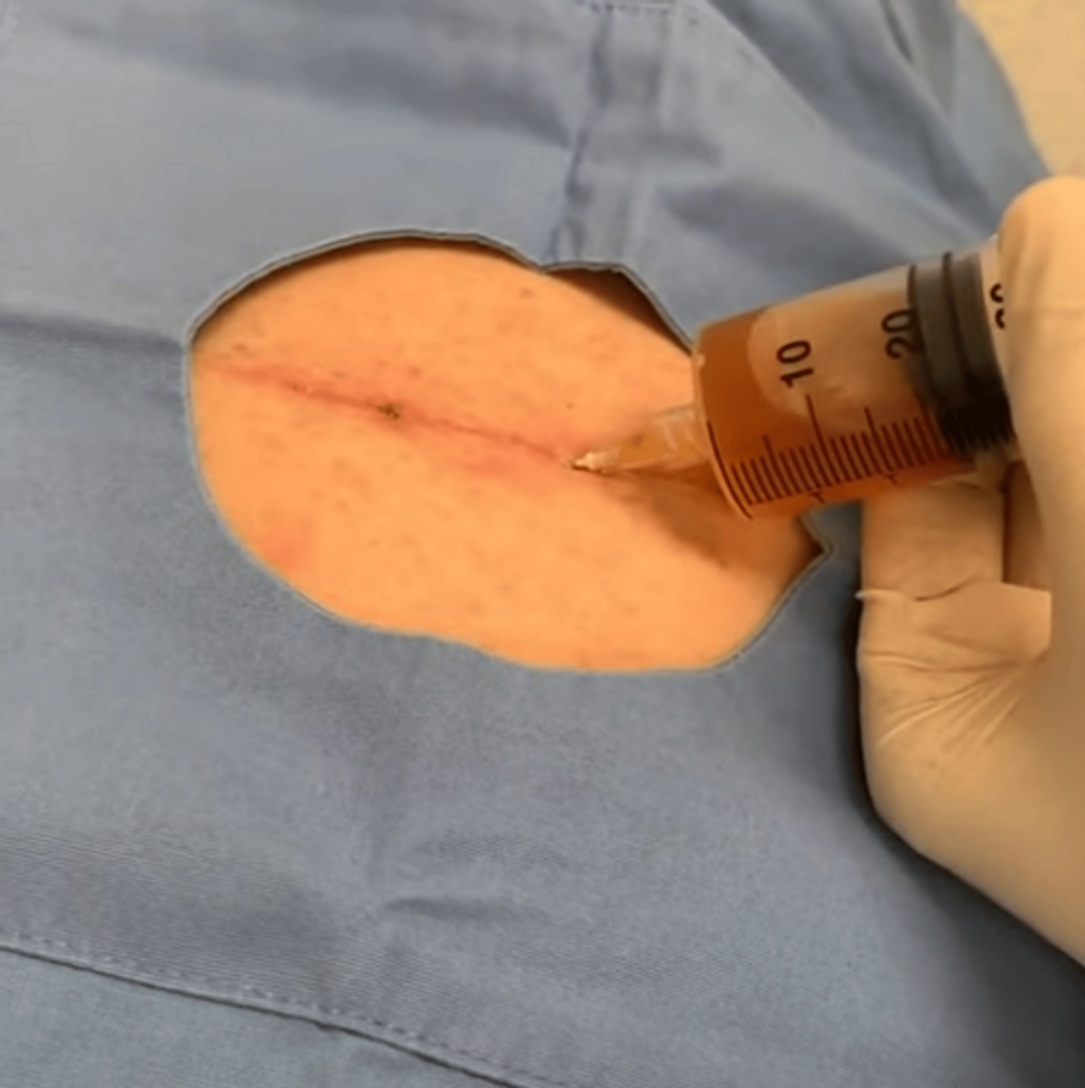
Aspiration of Seroma

**Figure 2:**
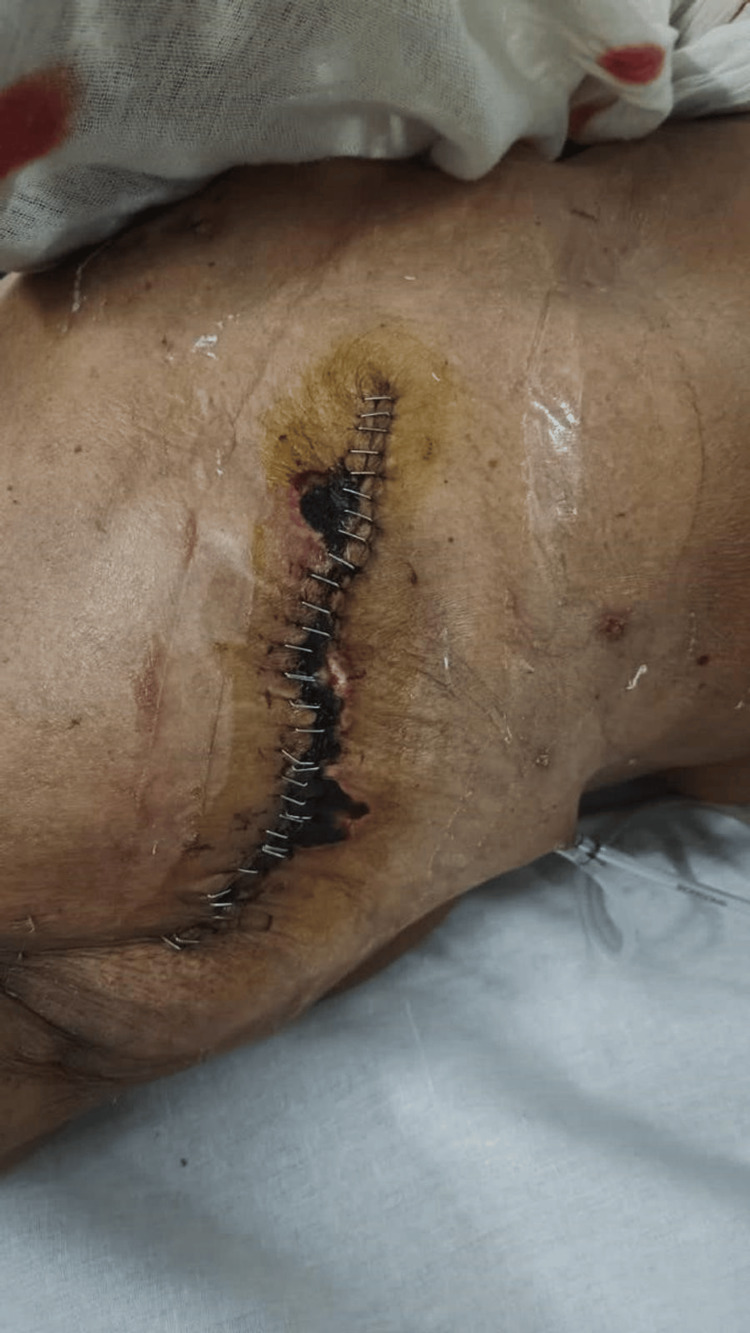
Marginal Flap Necrosis

Seroma was defined as a clinically appreciable fluid collection in the mastectomy or axillary dead space on POD 14, that required observation or aspiration (Figure 1). Flap necrosis was defined clinically as partial or full-thickness skin flap ischemia, identified on wound inspection and, when applicable, requiring debridement or further intervention (Figure 2). All data were recorded on a standardized questionnaire (Appendix 1), in the patient’s preferred language.

Subgroup and missing data handling

**Figure 3:**
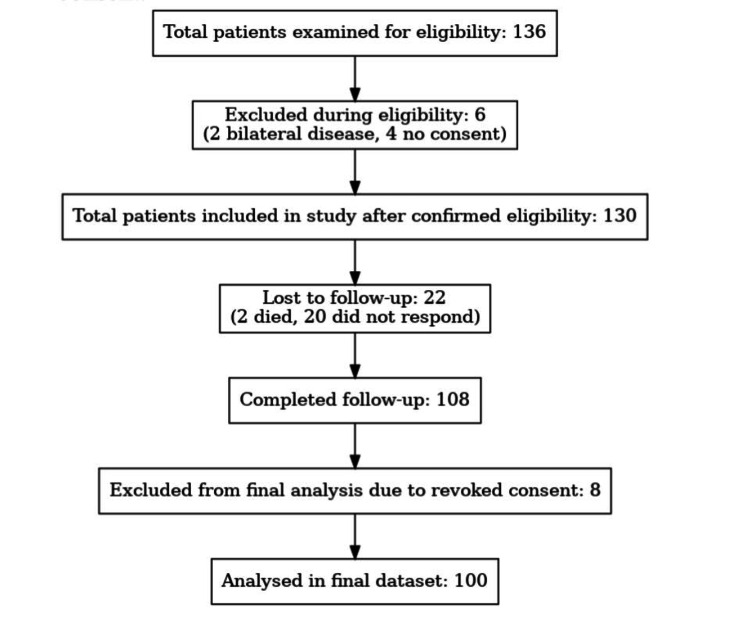
Total Patient Enrolment and Exclusion Flowchart

Prespecified subgroup analyses evaluated whether outcomes varied by age group (<30, 30-50, 51-70, and >70), BMI category (<25 vs. ≥25), lymph node status (positive vs. negative), and NACT exposure. Where appropriate, interaction terms were tested in regression models to explore effect modification (for example, age × BMI on seroma risk). The pattern of missing data was examined; missingness mechanisms were assessed and reported, and analyses were performed on complete cases with sensitivity checks. Any exclusions for incomplete follow-up are described in Figure 3 and in the Results section.

Bias reduction and standardization

To reduce bias and improve internal consistency, consecutive recruitment was used, and all surgeries were performed by the same surgical team following standardized operative and perioperative protocols. Clinical assessments were performed by designated investigators according to a predefined proforma. Follow-up visits were scheduled at standardized intervals (until discharge and at one to three months) to reduce recall bias and ensure outcome capture. Data were recorded on a predesigned case record form; missing data were actively monitored and documented. While the VAS is a validated instrument for pain assessment, the overall questionnaire used to capture clinical outcomes was developed for this study and did not undergo separate external psychometric validation (this limitation is acknowledged).

Sample size calculation

Sample size was calculated assuming an expected seroma incidence of ~30% (based on prior studies), 95% confidence (Z = 1.96), and a margin of error of ±10%. Using the formula for proportions, $n = \frac{Z^2 \times P \times (1 - P)}{E^2}$, yielded n ≈ 81; allowing for a 20% attrition rate produced a final target of 100 patients. This sample size was considered sufficient to estimate key frequencies (seroma and flap necrosis) with acceptable precision, and to detect moderate associations in subgroup analyses.

Statistical analysis

Data were analyzed using IBM SPSS Statistics for Windows, Version 29 (Released 2022; IBM Corp., Armonk, NY, USA). Continuous variables are presented as mean ± SD or median (IQR), depending on distribution; categorical variables are presented as frequencies and percentages. Associations between categorical variables were tested with the Chi-square test or Fisher’s exact test, as appropriate; comparisons of continuous variables used t-tests or Mann-Whitney U tests, depending on normality. Multivariable logistic regression was used, where appropriate, to adjust for potential confounders; interaction terms were tested for prespecified hypotheses. Statistical significance was defined as p < 0.05. All analyses were performed on complete-case datasets, and sensitivity analyses were performed when missing data exceeded prespecified thresholds.

## Results

Key findings

**Figure 4:**
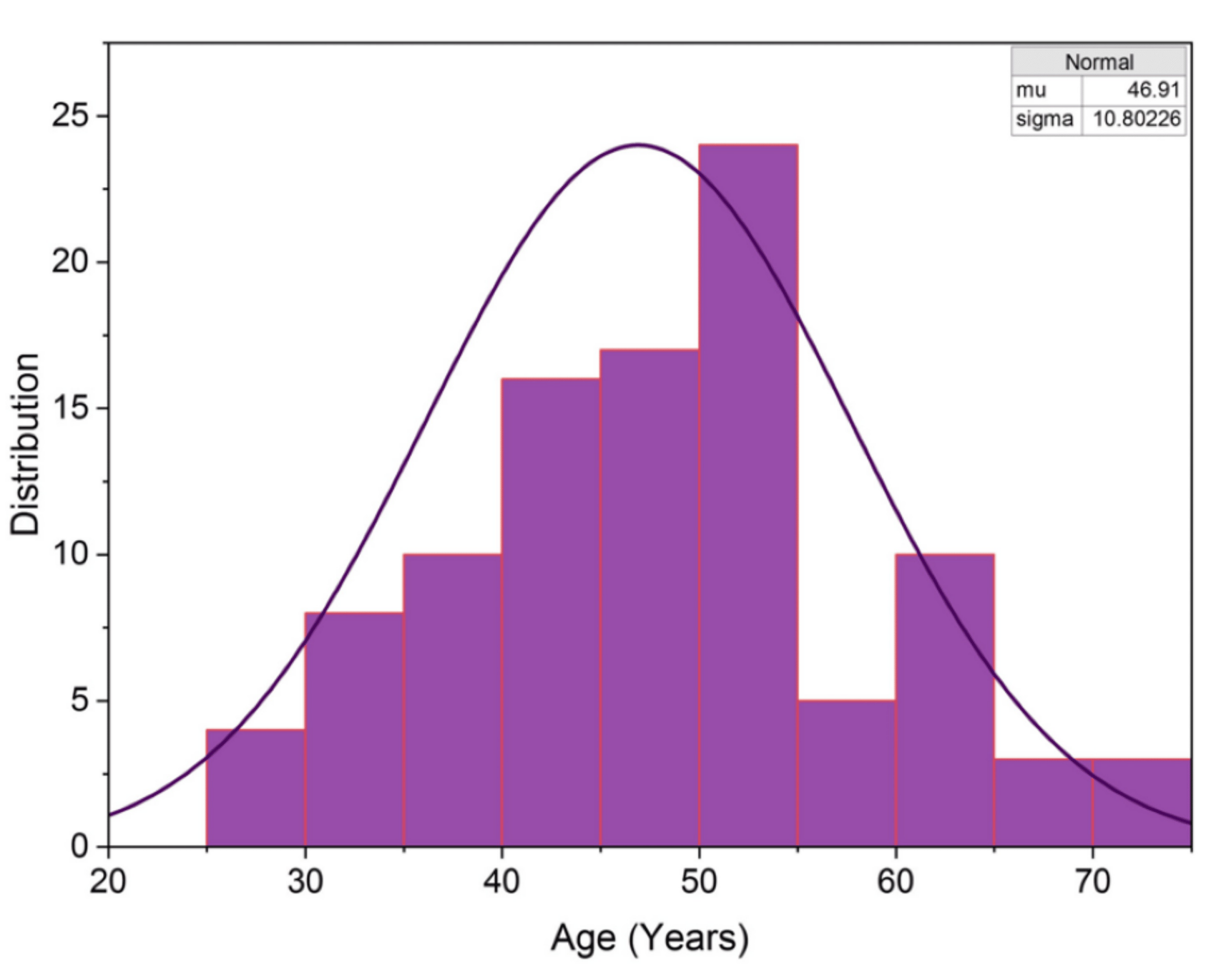
Distribution of the Participants in Terms of Age (Years) The observation made in this figure shows the skewness of the age distribution data as 0.153, with positive skewness, and kurtosis of -0.819, indicating a non-normal distribution. The majority of patients are within the 30-50 age group.

The study analyzed a total of 100 patients, and their demographic and clinical parameters were distributed across multiple categories. The age distribution of the participants revealed that the majority belonged to the 30-50 years age group, accounting for 57% (n = 57) of the total cohort. Figure 4 illustrates the skewness of the age distribution data, with a skewness value of 0.153 and kurtosis of -0.819, confirming a non-normal distribution. A slightly higher prevalence of right-sided breast cancer was observed, with 56% (n = 56) of patients presenting with disease on the right side. Positive lymph node status was recorded in 60% (n = 60) of cases, which often correlated with advanced disease stages. Most patients were in Stages IIA, IIIA, and IIB, comprising 32%, 31%, and 25% of the total cohort, respectively. In terms of BMI distribution, the majority of patients had a BMI under 25, accounting for 79% (n = 79), while 21% were classified as obese. NACT was administered to approximately half of the cohort, with 52% (n = 52) receiving it prior to surgery.

Postoperative complications, such as pain, seroma, and flap necrosis, were evaluated in detail. Pain severity showed a general decreasing trend from POD 7 to 90. Flap necrosis occurred in 20% (n = 20) of patients, while seroma formation was observed in 26% (n = 26) of the cohort. Pain severity on POD 7 demonstrated a significant association with age group (χ² = 18.487, p = 0.0299), indicating that different age groups experienced varying levels of pain. This finding underscores the importance of age-specific pain management strategies. NACT showed borderline significance in its association with pain severity on POD 7 (χ² = 6.320, p = 0.0970), suggesting that it might influence pain levels, although this association did not reach statistical significance at the 0.05 level. Other factors, including cancer side, lymph node status, disease stage, and BMI, showed no significant association with pain severity on POD 7 (Table 1).

**Table 1:** Chi-Square Test Results Showing Association Between Pain Levels on POD 7 and Various Clinical Parameters NACT: Neoadjuvant Chemotherapy

Parameter	Chi-Square Statistic	p-value	Significant Association
Age Group	18.487	0.0299 (<0.05)	Yes
Side	1.608	0.6577 (>0.05)	No
Lymph Node	0.167	0.9827 (>0.05)	No
Stage	18.64	0.4143 (>0.05)	No
BMI	1.847	0.6048 (>0.05)	No
NACT	6.32	0.097 (>0.05)	Borderline

Seroma formation was significantly associated with younger age groups (χ² = 7.894, p = 0.0483), highlighting an increased risk of seroma occurrence in younger patients. BMI demonstrated a strong association with seroma formation (χ² = 20.252, p < 0.001), with patients having a higher BMI being at greater risk. However, other factors, including side of breast cancer, lymph node involvement, disease stage, and NACT, did not show any significant association with seroma formation (Table 2).

**Table 2:** Chi-Square Test Results Showing Association Between Presence of Seroma on POD 14 and Various Clinical Parameters NACT: Neoadjuvant Chemotherapy

Parameter	Chi-Square Statistic	p-value	Significant Association
Age Group	7.894	0.0483 (<0.05)	Yes
Side	0.0	1.0 (>0.05)	No
Lymph Node Status	2.081	0.1491 (>0.05)	No
Stage	5.386	0.4953 (>0.05)	No
BMI	20.252	0.076 (<0.05)	Yes
NACT	0.816	0.3662 (>0.05)	No

The analysis of flap necrosis revealed a significant association with cancer stage (p = 0.0104), suggesting that patients with more advanced disease stages are at higher risk of developing flap necrosis. Other factors, such as age group, cancer side, lymph node status, BMI, and NACT, showed no statistically significant association with flap necrosis (Table 3).

**Table 3:** Chi-Square Test Results Showing Association Between Flap Necrosis and Various Clinical Parameters NACT: Neoadjuvant Chemotherapy

Parameter	Chi-Square Statistic	p-value	Significant Association
Age Group	5.112	0.1638 (>0.05)	No
Side	0.733	0.3919 (>0.05)	No
Lymph Node	1.628	0.202 (>0.05)	No
Stage	16.715	0.0104 (>0.05)	Yes
BMI	0.034	0.8539 (<0.05)	No
NACT	0.904	0.3417 (>0.05)	No

In assessing the character and nature of pain, a significant association was found between BMI and the nature of pain, with p < 0.001. This indicates that the type of pain experienced by patients is influenced by BMI. However, no significant association was observed between the character of pain (localized vs. radiating) and the clinical parameters, except for a statistically significant relationship with NACT intake (p = 0.003).

The impact of seroma and flap necrosis on postoperative pain levels was further analyzed. Patients with seroma formation experienced significantly higher pain levels on POD 15 (p = 0.0094), POD 30 (p = 0.0021), and POD 90 (p = 0.0305), suggesting a prolonged impact of seroma on pain outcomes. Conversely, no significant association was observed between flap necrosis and pain severity at any of the assessed time points, indicating that flap necrosis does not appear to influence long-term pain outcomes.

This comprehensive analysis highlights that specific patient demographics and clinical factors play a pivotal role in postoperative outcomes following MRM. Age, BMI, and disease stage emerged as key determinants influencing pain severity, seroma formation, and flap necrosis. Pain management strategies should account for patient age to ensure targeted interventions, while preventive measures for seroma formation should focus on younger patients and individuals with higher BMI. Although the borderline significance of NACT’s effect on pain warrants further investigation, the significant association between advanced cancer stage and flap necrosis emphasizes the need for tailored surgical planning and postoperative care in these high-risk groups. These findings provide valuable insights that can be utilized to optimize clinical care and improve recovery outcomes for patients undergoing MRM.

## Discussion

MRM remains a cornerstone of surgical management for breast cancer, particularly for patients presenting with advanced disease stages. While highly effective, MRM is associated with several postoperative complications that can affect recovery, delay adjuvant treatments, and lead to significant morbidity. This study provides an in-depth analysis of complications, such as seroma, wound infection, and flap necrosis, placing these findings in the context of the existing literature.

Seroma formation emerged as the most frequent postoperative complication, affecting 26% of patients in this study. This result aligns closely with the 25.8% incidence reported by Ali et al. [16] in Muhammad Teaching Hospital, Peshawar. However, higher rates were noted in the study by Obadiel et al. [17] at Al-Gomhori Teaching Hospital, Yemen, which recorded an incidence of 44%. Schou Bredal et al. [18] highlighted the association of seroma formation with factors like smoking, hypertension, and neoadjuvant chemotherapy, which could explain variations across studies. Other studies, such as those by Chandrakar and Shinde [19] and Dahri et al. [20], reported incidences of 26.82% and 33.3%, respectively. Differences in demographics, surgical techniques, and perioperative care may underlie these variations. Importantly, higher BMI was strongly associated with seroma formation in this and previous studies, as corroborated by Shaikh et al. [21]. To mitigate seroma risk, strategies like optimal drain placement, prolonged drainage periods, and the use of tissue adhesives have been recommended by multiple researchers, including Schou Bredal et al. [18] and Chandrakar and Shinde [19].

Wound infection was observed in 24.39% of patients, a rate similar to that reported by Chandrakar and Shinde [19]. However, this is significantly higher than the rates reported by Dahri et al. [20] at 10% and Shaikh et al. [22] at 4.5%. The elevated infection rate in this study may reflect regional disparities in infection control practices and resource constraints. Contributing factors, such as malnutrition, inadequate hygiene, and delayed wound care, are particularly relevant in low-resource settings. Obadiel et al. [17] recorded an even higher wound infection rate of 28%, further emphasizing the impact of infection control measures on outcomes. Diabetes, hypertension, and elevated BMI have been identified as key risk factors for wound infections. The need for stringent perioperative practices, including prophylactic antibiotics, meticulous surgical technique, and rigorous postoperative wound care, has been consistently emphasized in the literature [16,18,20].

Flap necrosis, although less common, is a significant complication that adversely affects recovery and wound healing. This study reported a flap necrosis incidence of 20%, which is considerably higher than the 2.44% noted by Chandrakar and Shinde [19] and the 2.6% documented by Dahri et al. [20]. Similarly, Obadiel et al. [17] observed a much lower rate of 2%, suggesting that factors like advanced disease stages, comorbidities, and the use of diathermy may influence outcomes. Smoking, poor nutritional status, and diabetes have been consistently identified as major contributors to flap necrosis. The inclusion of patients with advanced disease stages in this study may explain the higher incidence. While diathermy is efficient, its thermal effects on tissue viability have been associated with increased necrosis rates. Improved surgical techniques, such as careful flap tension management and judicious use of diathermy, are critical for reducing necrosis risks, as highlighted by Chandrakar and Shinde [19].

Postoperative pain remains a significant concern, particularly in younger patients and those undergoing extensive procedures like ALND. This study's findings are consistent with those of Schou Bredal et al. [18], who emphasized the psychosocial and medical burden of post-surgical pain. Effective pain management strategies, such as multimodal analgesia, early physical therapy, and psychological support, are essential for improving patient outcomes. Chandrakar and Shinde [19] also highlighted the importance of proactive pain management in surgical planning and postoperative care.

This study underscores the multifactorial nature of postoperative complications following MRM. Advanced disease stages, necessitating extensive surgical interventions, predispose patients to higher complication rates. Similar findings have been reported in Obadiel et al.’s study [17], which also highlighted the role of advanced staging in increasing morbidity. BMI emerged as a critical factor influencing outcomes, with higher BMI associated with increased risks of seroma and wound infections, consistent with observations by Shaikh et al. [21]. These findings emphasize the importance of individualized patient assessment and tailored perioperative care.

Practical preventive measures

Based on our findings and existing literature, several practical steps may reduce postoperative morbidity after MRM: (1) meticulous flap handling and preservation of an appropriate subcutaneous thickness to preserve perfusion; (2) judicious use of electrocautery and minimization of thermal damage; (3) routine placement and appropriate management of suction drains, with consideration of prolonged drainage in high-risk patients; (4) use of quilting sutures or tissue adhesives, where applicable, to obliterate dead space; (5) optimization of modifiable patient risk factors preoperatively (smoking cessation, glycemic control, nutritional support, and weight management); and (6) early, structured physiotherapy and multimodal analgesia to improve recovery and reduce chronic pain risk. These measures should be evaluated prospectively in larger, preferably multicenter, cohorts.

Strengths and limitations

This study’s prospective cohort design and consecutive patient recruitment reduced recall and selection bias and permitted temporal assessment of postoperative outcomes after MRM. The use of a standardized data proforma, predefined clinical criteria for seroma and flap necrosis, scheduled follow-up visits, and consistent assessments by the same surgical team improved internal consistency. Prespecified subgroup and interaction analyses (age, BMI, lymph-node status, and NACT) enhanced clinical interpretability for tertiary-care settings.

Important limitations remain. The single-center design and modest sample size (n = 100) limit external generalizability. We limited operator variability by employing a consultant-led surgical team with standardized operative protocols; nonetheless, institutional practice patterns and surgeon experience may still limit external generalizability. Although the VAS is a validated tool for pain, the broader questionnaire used in this study did not undergo external psychometric validation, which may affect reproducibility. The observational design identifies associations but cannot establish causality; residual confounding is possible despite multivariable checks. Finally, surgical technique and perioperative resources vary across centers and may influence complication rates. We recommend multicenter studies with larger cohorts and validated patient-reported instruments to confirm and extend these findings.

In this prospective cohort of patients undergoing MRM, seroma formation (26%), flap necrosis (20%), and postoperative pain were common complications associated with patient factors such as younger age, higher BMI, and advanced disease stage. These associations identify higher-risk groups that may benefit from targeted perioperative strategies. As an observational study, our results are hypothesis-generating rather than causal; larger multicenter studies and interventional trials are needed to evaluate the effectiveness of specific preventive and therapeutic measures (e.g., quilting sutures, optimized drain protocols, and flap perfusion monitoring) on reducing complication rates and improving the timeliness of adjuvant therapy.

## Conclusions

This prospective cohort highlights that seroma formation, wound infection, flap necrosis, and postoperative pain remain important sources of morbidity after MRM, with seroma in 26% and flap necrosis in 20% of cases. Associations with younger age, elevated BMI, and advanced stage suggest identifiable high-risk groups who may benefit from focused perioperative interventions.

Practical measures, including meticulous flap handling and preservation of subcutaneous thickness, judicious electrocautery use, optimized drain protocols (and consideration of quilting sutures or tissue adhesives), preoperative optimization of comorbidities, and age-tailored multimodal analgesia with early physiotherapy, should be considered and evaluated prospectively. Given the observational design, these findings are hypothesis-generating; multicenter studies and randomized or pragmatic trials are required to establish causality and to standardize effective preventive protocols that preserve oncological timelines and improve patient outcomes.

## Appendices

Appendix 1

Study Title: Analysis of Seroma Formation, Flap Necrosis, and Postoperative Pain Following Modified Radical Mastectomy in Patients With Breast Cancer: A Prospective Study

(1) Details

Name                                ________________________

Age/Sex                            ________________________

Mobile Number                ________________________

Occupation                      ________________________

Address                           ________________________

Marital Status                  ________________________

Height (cm)                      ________________________

Weight (kg)                      ________________________

BMI (kg/m²)                      ________________________

Socioeconomic Status     ________________________

Date of Admission            ________________________

Hospital Registration No. ________________________

Date of Surgery                ________________________

(2) Clinical History

Chief Complaints: ____________________________________________

History of Present Illness (HOPI): ____________________________

Family History: ☐ Positive ☐ Negative

Habits: ☐ Smoking ☐ Alcohol ☐ None

Parity: ___________________

Comorbidities: ☐ Diabetes ☐ Hypertension ☐ Tuberculosis ☐ None

(3) General Physical Examination

Vital Signs:

Parameter           Finding

BP                        _______ mmHg

Pulse                   _______ /min

Respiratory Rate _______ /min

Temperature        _______ °C

General Condition:

☐ Pallor ☐ Icterus ☐ Cyanosis ☐ Oedema ☐ Clubbing ☐ Lymphadenopathy

(4) Systemic Examination

System               Findings

CNS                    __________________________

CVS                    __________________________

Respiratory        __________________________

GIT                     __________________________

(5) Disease Characteristics

Parameter                                      Details

Diagnosis                                        ______________________________________

TNM Stage                                     ☐ I ☐ IIA ☐ IIB ☐ IIIA ☐ IIIB ☐ IV

Histological Grade                         ☐ Well ☐ Moderate ☐ Poorly Differentiated

Neoadjuvant Chemotherapy         ☐ Yes ☐ No

Radiotherapy                                  ☐ Yes ☐ No

Pathological Stage                         __________________________

Receptor Status                             ER: ☐ + ☐ - PR: ☐ + ☐ - HER2-neu: ☐ + ☐ -

(6) Preoperative Assessment

Parameter                                                       Finding

Tumor Assessment (size, quadrant)            __________________________

Axillary Lymph Node Assessment                __________________________

Metastasis Evaluation (Imaging)                   __________________________

(7) Intraoperative Findings

Parameter                                                      Observation

Flap Thickness                                              __________________________

Type of Incision                                             __________________________

Placement of Drain                                       ☐ Axilla ☐ Flap ☐ Both

Number of Drains                                          ______

Tension on Flap during Closure                   ☐ None ☐ Mild ☐ Moderate ☐ Severe

Duration of Surgery (minutes)                      ______

(8) Postoperative Evaluation

A. Flap Necrosis

Parameter                                  Finding

Distribution                                ☐ Marginal ☐ Whole Flap

Interval of Presentation            __________ POD

Area Involved (cm²)                  __________

Intervention Done                     ☐ Conservative ☐ Debridement ☐ Secondary Closure

B. Pain Assessment

Visual Analog Scale (VAS: 0-10)

POD      VAS Score  Pain Severity  Character (Localized/Radiating)  Nature (Dull/Sharp)

POD 7        ___                ___                    ___                                                ___

POD15       ___                ___                    ___                                                ___

POD30      ___                ___                    ___                                                ___

POD 60     ___                ___                    ___                                                ___

POD 90     ___                ___                    ___                                                ___

Analgesia Details:

Drug                         Dose   Frequency   Duration   Rescue Opioid Needed

Paracetamol            ___        ___                ___            ☐ Yes ☐ No

NSAID                      ___        ___                ___            ☐ Yes ☐ No

Opioid                      ___        ___                ___            ☐ Yes ☐ No

C. Seroma

Parameter                                                                Entry

Drain Duration (days)                                               ___

Average Drain Output (mL/day)                               ___

Clinically Appreciable Seroma on POD 14               ☐ Yes ☐ No

Aspiration Performed                                                ☐ Yes ☐ No   Volume: ___ mL

Complications (Infection/Recurrence)                      __________________________

Duration of Resolution                                               __________________________

(Assessment of seroma done clinically)

(9) Investigations

Test                                                   Finding

Hemoglobin                                      _______ g/dL

TLC/Platelets                                    _______ /mm³

Liver Function Test                           __________________

Serum Albumin                                  _______ g/dL

FNAC / Biopsy                                   __________________

HRCT Chest                                       __________________

(10) Follow-Up Evaluation

Visit                                          Findings                           Remarks

Discharge                            ______________                    ____________

1 Month                                ______________                   ______________

3 Months                              ______________                   ______________

(11) Investigator Details

Field                                                      Entry

Investigator Name                                __________________________

Designation                                           __________________________

Signature                                               __________________________

Date                                                       __________________________

Notes:

(i) Pain assessment based on validated VAS (0-10).

(ii) Seroma and flap necrosis are defined clinically per study protocol.

(iii) Data collected in English/Hindi based on patient preference.
